# APOE and Cerebral Microbleeds: Insights Into Causal Proteins and Therapeutic Targets

**DOI:** 10.1002/brb3.71382

**Published:** 2026-06-19

**Authors:** Lei Yang, Jian‐Lan Zhao, Jie Song, Lin‐Hui Chen, Chun Yu, Qiang Yuan, Gang Wu, Jin Hu, Mei‐Hua Wang

**Affiliations:** ^1^ Department of Neurosurgery & Neurocritical care, Huashan Hospital Fudan University Shanghai China; ^2^ Department of Neurosurgery, National Center for Neurological Disorders, Neurosurgical Institute of Fudan University, Shanghai Clinical Medical Center of Neurosurgery, Shanghai Key Laboratory of Brain Function and Restoration and Neural Regeneration, Huashan Hospital Fudan University Shanghai China

**Keywords:** APOE, cis‐pQTLs, cerebral microbleeds, lifestyle factors, Mendelian randomization, protein–protein interaction, therapeutic targets

## Abstract

**Background:**

Cerebral microbleeds (CMBs) are small chronic brain hemorrhages that can be linked to cognitive decline and stroke risk. Understanding the underlying genetic and proteomic mechanisms can enhance therapeutic interventions. This study aimed to explore the relationship between circulating proteins (cis‐pQTLs) and CMBs using Mendelian randomization (MR) and colocalization analysis, along with protein–protein interaction (PPI) network construction and the evaluation of lifestyle factors influencing CMB‐related proteins.

**Methods:**

A two‐sample MR approach was employed to investigate potential causal relationships between protein levels and CMB risk using cis‐pQTLs as instrumental variables. Colocalization analysis was conducted to assess whether the same genetic variants influence both circulating proteins and CMB susceptibility. Additionally, a PPI network was built to prioritize therapeutic targets, and drug efficacy was evaluated using known protein targets. A systematic MR analysis examined the impact of 17 healthy lifestyle factors on CMB‐related proteins.

**Results:**

MR analysis revealed an association between 79 plasma proteins and CMBs, with one protein remaining significant after false discovery rate correction. Colocalization analysis identified APOE as a causal protein for CMBs, supported by a strong posterior probability (PP4 = 0.996). PPI network analysis highlighted the potential bleeding risks of antithrombotic medications in CMB pathogenesis and Rosuvastatin. Furthermore, MR analysis showed that dietary factors, particularly cooked vegetable intake, were associated with APOE‐related pathways, though these findings warrant cautious interpretation regarding lifestyle modifications.

**Conclusions:**

This study provides evidence for a causal relationship between circulating proteins and CMBs, with APOE identified as a key factor. The identification of coagulation‐related proteins highlights the potential risks of antithrombotic medications in CMB pathogenesis. Lifestyle factors may influence protein‐mediated CMB risk, suggesting avenues for future research into gene–environment interactions.

## Graphical abstract

This study identifies APOE as a key causal protein for cerebral microbleeds (CMBs). By integrating Mendelian randomization (MR) and colocalization analysis of the plasma proteome from UKB‐PPP and deCODE datasets, we provide genetic evidence that circulating APOE levels are causally linked to CMB susceptibility. Furthermore, protein–protein interaction (PPI) analysis highlights the shared pathways between APOE and coagulation‐related drug targets, while dietary habits, specifically cooked vegetable intake, were found to influence APOE‐mediated risk.

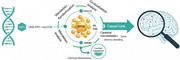

## 1 Introduction

Cerebral microbleeds (CMBs) represent a critical yet often underappreciated aspect of cerebrovascular pathology. These small, chronic brain hemorrhages, typically less than 10 mm in diameter, are increasingly recognized as important markers of underlying vascular vulnerability and potential harbingers of more severe cerebrovascular events (Greenberg et al. 2009; Charidimou et al. 2017). The clinical significance of CMBs extends beyond their size, as they have been associated with an elevated risk of cognitive decline, stroke, and other neurological disorders (J. M. Wardlaw et al. 2013; Shams et al. 2015). As our population ages and neuroimaging techniques continue to advance, the prevalence and importance of CMBs in both clinical practice and research settings have grown substantially (Poels et al. 2011). The etiology of CMBs is multifaceted, involving a complex interplay of genetic, environmental, and vascular factors. Hypertensive arteriopathy and cerebral amyloid angiopathy are two primary pathological processes linked to the formation of CMBs, each with distinct spatial distribution patterns within the brain (Jolink et al. 2023; Shim et al. 2015). Hypertension‐related CMBs tend to occur in deep brain structures and the brainstem, while those associated with cerebral amyloid angiopathy are more commonly found in lobar regions (Akoudad et al. 2015). This heterogeneity in location and underlying pathology underscores the need for a nuanced understanding of CMB formation and progression.

Despite the growing recognition of CMBs as important biomarkers of cerebrovascular health, there remains a significant gap in our understanding of the molecular mechanisms underlying their formation and the genetic factors that may predispose individuals to develop these lesions (Tan and Markus 2015). Recent advances in genomics and proteomics have opened new avenues for exploring these questions, offering the potential to identify novel targets for therapeutic intervention and prevention strategies (Söderholm et al. 2019). One particularly promising area of research involves the study of circulating proteins and their relationship to CMB risk. Proteins play crucial roles in various biological processes, including those involved in vascular integrity, inflammation, and blood–brain barrier function (Dutta et al. 2012). By examining the genetic determinants of protein levels (known as protein quantitative trait loci [pQTLs]) and their association with CMBs, we can gain insights into the causal pathways that may contribute to CMB formation.

The advent of Mendelian randomization (MR) techniques has provided a powerful tool for investigating potential causal relationships between circulating proteins and disease outcomes. This approach leverages genetic variants as instrumental variables to mitigate confounding and reverse causation, two common limitations in observational studies (Davey Smith and Hemani 2014; Emdin et al. 2017). By applying MR to the study of CMBs, we can more confidently identify proteins that may play a causal role in their development, potentially uncovering new therapeutic targets. Furthermore, the integration of MR with colocalization analysis offers an additional layer of evidence for causality. Colocalization analysis examines whether the genetic variants associated with protein levels and those associated with CMB risk are likely to represent the same causal variant (Raffield et al. 2020). This approach can help distinguish between situations where a protein is merely a biomarker of CMB risk and those where it may be directly involved in the causal pathway. Recent studies have successfully utilized analogous MR frameworks to explore causal molecular mechanisms across complex diseases, such as the roles of immune cells and gut microbiota in various conditions (Zhang et al. 2021; Y. Liu et al. 2025; Nian et al. 2025; R. Liu et al. 2024; Hu et al. 2024)

The potential clinical implications of identifying causal proteins for CMBs are significant. Such discoveries could lead to the development of novel biomarkers for early detection and risk stratification, as well as new therapeutic targets for preventing or mitigating the progression of CMBs (Evangelou and Ioannidis 2013). Given the association between CMBs and various neurological disorders, including cognitive decline and stroke, interventions targeting these proteins could have far‐reaching effects on cerebrovascular health (J. Wardlaw et al. 2017). In addition to exploring the genetic and proteomic underpinnings of CMBs, it is crucial to consider the role of modifiable lifestyle factors in their development and progression. Understanding how dietary habits, physical activity, and other lifestyle choices influence the levels of CMB‐associated proteins could provide valuable insights for developing preventive strategies and public health interventions.

The construction of protein–protein interaction (PPI) networks represents another valuable approach in this research (Kim et al. 2022). By mapping the interactions between CMB‐associated proteins and known drug targets, we can potentially identify existing medications that might be repurposed for CMB prevention or treatment. This strategy could significantly accelerate the translation of research findings into clinical practice, offering new hope for patients at risk of CMB‐related complications. In light of these considerations, this study aims to comprehensively investigate the relationship between circulating proteins and CMBs using a multi‐faceted approach. By combining two‐sample MR, colocalization analysis, PPI network construction, and the evaluation of lifestyle factors, we seek to (1) identify circulating proteins causally associated with CMB risk, (2) validate these associations through colocalization analysis, (3) explore potential therapeutic targets through PPI network analysis and drug target evaluation, and (4) assess the impact of lifestyle factors on CMB‐related proteins.

This integrated approach not only promises to enhance our understanding of the molecular mechanisms underlying CMB formation but also has the potential to identify novel therapeutic strategies and preventive measures. By bridging the gap between genetic susceptibility, protein function, and modifiable risk factors, this research aims to contribute significantly to the field of cerebrovascular health and pave the way for more personalized and effective interventions for individuals at risk of CMBs and their associated complications.

## 2 Materials and Methods

### 2.1 Study Design

This study initially investigated the association between circulating proteins (cis‐pQTL) and CMBs using two‐sample MR analysis. This method was employed to explore potential causal relationships between protein levels and the risk of CMBs. To further confirm the causality, colocalization analysis was conducted to determine whether the same genetic variants influence both circulating proteins and CMB susceptibility, strengthening the evidence for a causal link.

**FIGURE 1:**
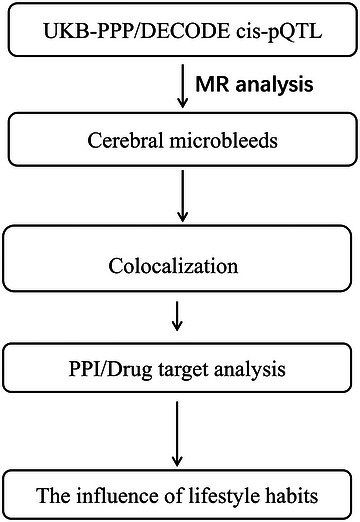
This flowchart illustrates the main analytical steps of the study. First, a two‐sample Mendelian randomization analysis was conducted to explore the association between circulating proteins (cis‐pQTLs) and cerebral microbleeds. Next, a colocalization analysis will be conducted to verify the causal relationship between circulating proteins and susceptibility to cerebral microbleeds. Subsequently, protein–protein interaction analysis was conducted to identify potential therapeutic targets. Finally, a systematic Mendelian randomization analysis was conducted to evaluate the impact of lifestyle factors on Cerebral microbleeds‐related proteins.

Next, PPI network analysis was performed to identify possible interactions between relevant proteins. This analysis, combined with drug efficacy evaluations, allowed for the prioritization of proteins that could serve as therapeutic targets. Finally, a systematic MR approach was used to analyze the relationship between healthy lifestyle factors and CMB‐related proteins, with the goal of identifying proteins that may be modifiable through lifestyle interventions (Figure 1).

### 2.2 Data Sources

We conducted a genome‐wide association study (GWAS) using data from 35,559 Icelandic individuals, analyzing 4907 aptamers on the SomaScan platform (Emilsson et al. 2018). This study identified 28,191 genetic associations, with data derived from two primary sources: the Icelandic Cancer Project (52% of participants) and various genetic projects at deCODE Genetics (48% of participants). Using recursive conditional analysis, we highlighted the most significant genetic variations in each region (± 1 Mb), identifying 18,084 sentinel pQTLs and 10,107 secondary pQTLs. Our findings replicated 83% of the pQTLs from the INTERVAL study (SomaScan) and 64% from the SCALLOP consortium (Olink), demonstrating the strong overlap and reliability of these genetic associations in identifying disease mechanisms via proteins (PMID: 34857953).

Additionally, we utilized data from the UKB‐PPP Pharmaceutical Proteomics Project, which examined plasma proteomic profiles in 54,219 participants from the UK Biobank (Sun et al. 2018). This comprehensive analysis provided new insights into protein–disease relationships, offering both technical validation and predictive models based on demographic and health data. The study mapped 2923 proteins, uncovering 14,287 genetic associations, 81% of which were previously unreported, including those specific to non‐European ancestries. As the study predicted, further advances in sample size and mass spectrometry will accelerate pQTL discovery. The results also emphasized the impact of pQTLs on ligand–receptor interactions and cytokine pathways, with clear implications for drug discovery, including genetic insights into targets like PCSK9 and gene–protein perturbations related to COVID‐19 susceptibility (PMID: 37794186).

Data on CMBs were sourced from a specialized study (PMID: 32913026), and lifestyle data were drawn from the IEU openGWAS project.

### 2.3 Acquisition of Cis‐pqtl

All cis‐pQTL data used in this study were obtained directly from their original sources, as detailed in Section 2. This ensures the integrity and reliability of the genetic associations analyzed, providing a solid foundation for the subsequent analyses.

### 2.4 MR Analysis

Genetic variants (pQTLs) significantly associated with protein levels were obtained from GWAS. These pQTLs include both cis‐pQTLs, located near the coding genes they regulate, and trans‐pQTLs, which are situated farther from the genes (Zheng et al. 2020). For the MR analysis, cis‐pQTLs were prioritized as the primary instrumental variables due to their closer proximity to the genes, which typically results in stronger and more direct associations with protein levels (Yaghootkar et al. 2020).

In the statistical analysis, the TwoSampleMR package in R was employed to perform the MR analysis between cis‐pQTLs and CMBs. For cis‐pQTLs associated with only one single‐nucleotide polymorphism (SNP), the Wald ratio method was used as the primary estimate (Burgess and Thompson 2015). For cis‐pQTLs with multiple SNPs, inverse‐variance weighted results were prioritized, with additional methods applied as complementary approaches to ensure robustness (Bowden et al. 2016).

### 2.5 Colocalization Analysis

To assess the colocalization of genetic variants within candidate gene regions and their potential effects on different phenotypes, such as disease traits and gene expression levels, we performed a colocalization analysis (Giambartolomei et al. 2014). This analysis aims to determine whether the same genetic variant influences two distinct phenotypes, providing valuable insights into potential causal relationships.

The colocalization analysis was carried out using the coloc R package. This method calculates five posterior probabilities (PPs) corresponding to different hypotheses: PP0 assumes no association in the region, PP1 assumes an association for the first trait but not the second, PP2 assumes an association for the second trait but not the first, PP3 assumes that both traits are associated but due to different genetic variants, and PP4 assumes both traits are associated and share the same causal variant (Wallace 2020). Our primary focus was on PP4, with a value greater than 0.8 considered strong evidence of colocalization, indicating a shared causal variant between the phenotypes.

### 2.6 Evaluation of Medicinal Properties of Drugs and PPI

We began by using DrugBank to compile a list of known targets for drugs commonly used in the treatment of CMBs (Wishart and Wu 2016). This allowed us to assess the potential therapeutic relevance of proteins that had been identified with strong evidence as viable targets for CMB intervention. Proteins targeted by approved drugs or those in clinical or experimental stages were classified as potential drug targets, and detailed drug information for these proteins was documented.

Next, we constructed a PPI network using the STRING database to further explore the relationships between the identified drug targets and the newly predicted protein targets. This step was crucial in determining whether existing drug targets could interact with, or influence, our newly predicted targets, thereby providing insights into the broader therapeutic landscape for CMBs.

### 2.7 The Impact of Lifestyle on Predicting Protein Targets

Additionally, we performed MR analysis to explore the impact of lifestyle factors on proteins related to CMBs. This analysis aimed to identify which proteins associated with CMBs could potentially be modified through lifestyle interventions. A total of 17 lifestyle factors (listed in Supporting Information S1) were evaluated for their association with these targeted proteins.

The MR methodology used for this analysis followed the same approach as described for the proteome‐wide analysis, ensuring consistency and robustness. All statistical analyses were conducted using R software version 4.4.2, providing a reliable framework for evaluating the causal links between lifestyle factors and CMBs‐related proteins (Y. Liu et al. 2019).

## 3 Result

### 3.1 Mendelian Randomization Analysis of Plasma Proteins and Cerebral Microbleeds

**FIGURE 2:**
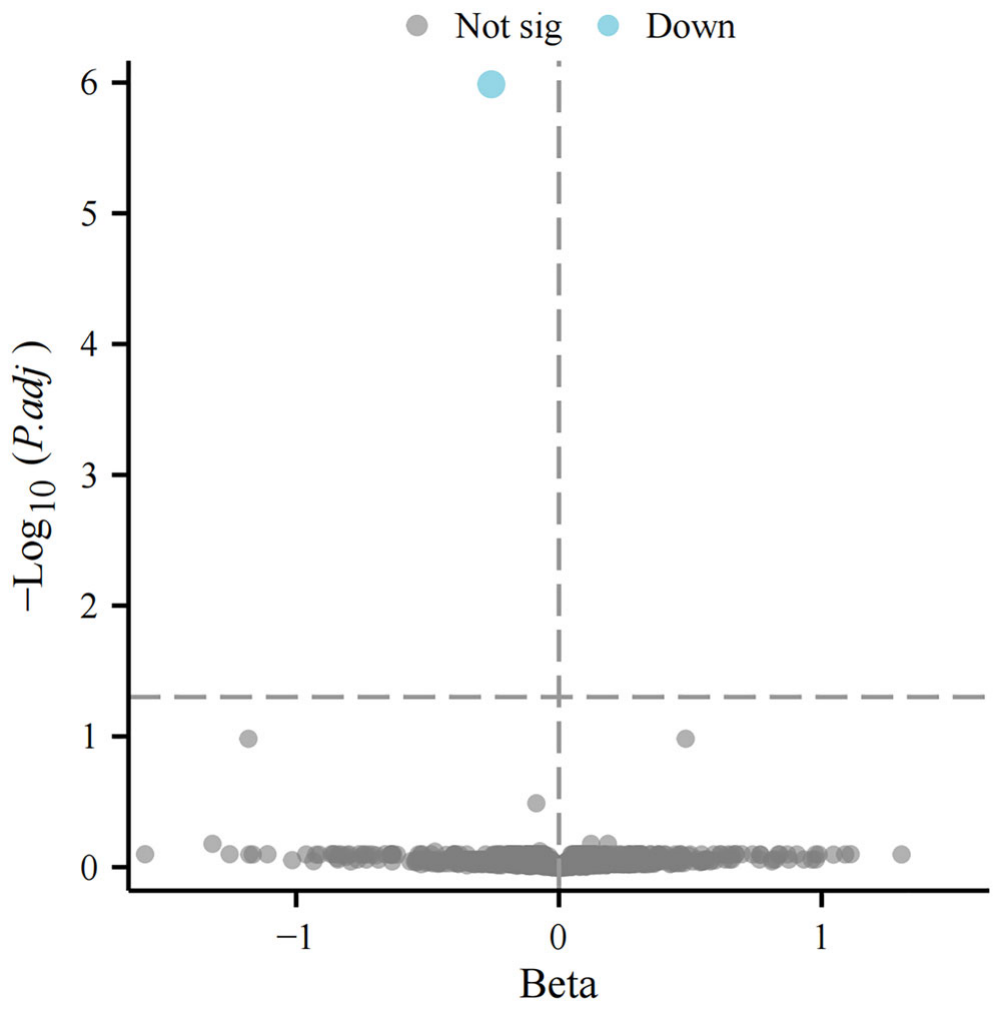
Volcano plot illustrating the number of circulating plasma proteins with significant causal associations with cerebral microbleeds after false discovery rate (FDR) correction. Blue dots represent proteins with significant downregulation (FDR < 0.05), while gray dots indicate nonsignificant results.

**FIGURE 3:**
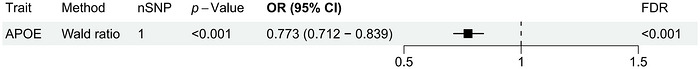
Forest plot showing the causal relationship between APOE protein levels and cerebral microbleeds after FDR correction. The odds ratio (OR) and 95% confidence interval (CI) are presented for each association, with APOE showing a statistically significant protective effect (OR < 1, FDR < 0.05).

In the initial analysis of cis‐pQTLs and CMBs using UKB‐PPP data, we identified an association between 79 plasma proteins and CMBs at a nominal significance level (*p* < 0.05). However, after adjusting for the false discovery rate (FDR), only one protein maintained a statistically significant association with CMBs (FDR < 0.05, as shown in Figures 2 and 3, Supporting Information S2). This highlights the necessity of FDR correction in large‐scale proteomic studies to minimize the risk of false positives. We then validated these findings using deCODE's cis‐pQTL data, which yielded consistent results, further supporting the robustness of this protein association (Supporting Information S3).

### 3.2 Proteins Were Validated Through Colocalization Evidence

**FIGURE 4:**
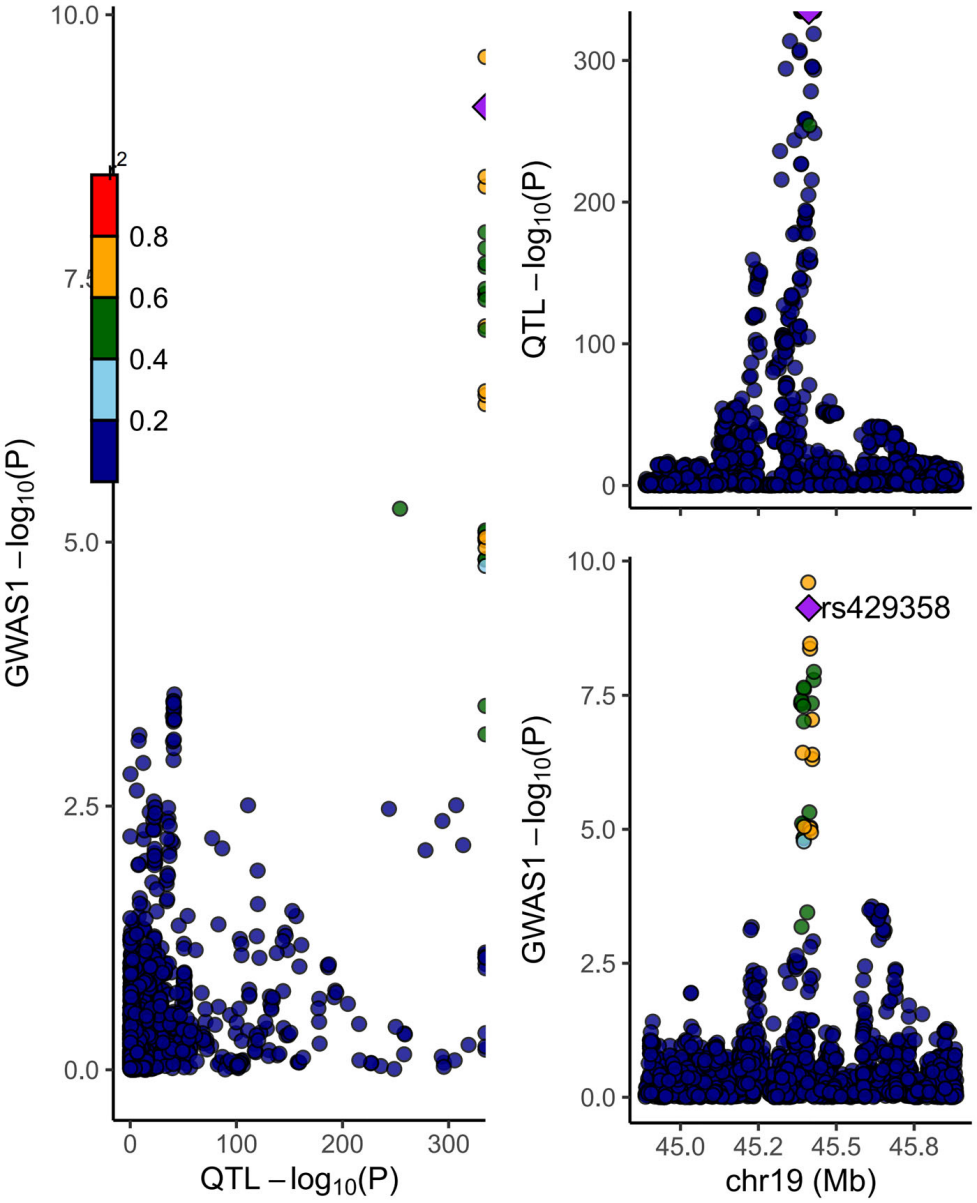
Colocalization analysis of APOE and cerebral microbleeds using UKB‐PPP data. The left panel compares the strength of associations from the GWAS and QTL datasets, highlighting colocalization points with significant signals. The top right panel shows the QTL signals along chromosome 19, and the bottom right panel focuses on the GWAS signals in the same region. The purple diamond marks the rs429358 SNP, strongly associated with both APOE and cerebral microbleeds. Color intensity represents the strength of association, with a posterior probability of colocalization (PP4) of 0.996, suggesting a high likelihood that APOE is causally linked to cerebral microbleeds.

Using data from UKB‐PPP, we found strong colocalization evidence for the APOE protein across multiple genomic windows, with a posterior probability (PP4) of 0.996, indicating a high likelihood that APOE is a causal factor for CMBs (Figure 4). To rule out the possibility of reverse causality, we conducted the Steiger test, which assesses the directionality of the association. The results confirmed that all SNPs involved had no issues with directionality, reinforcing the robustness of the causal link between APOE and CMBs (Supporting Information S4).

### 3.3 Drug Targets and PPI Network Construction

**FIGURE 5:**
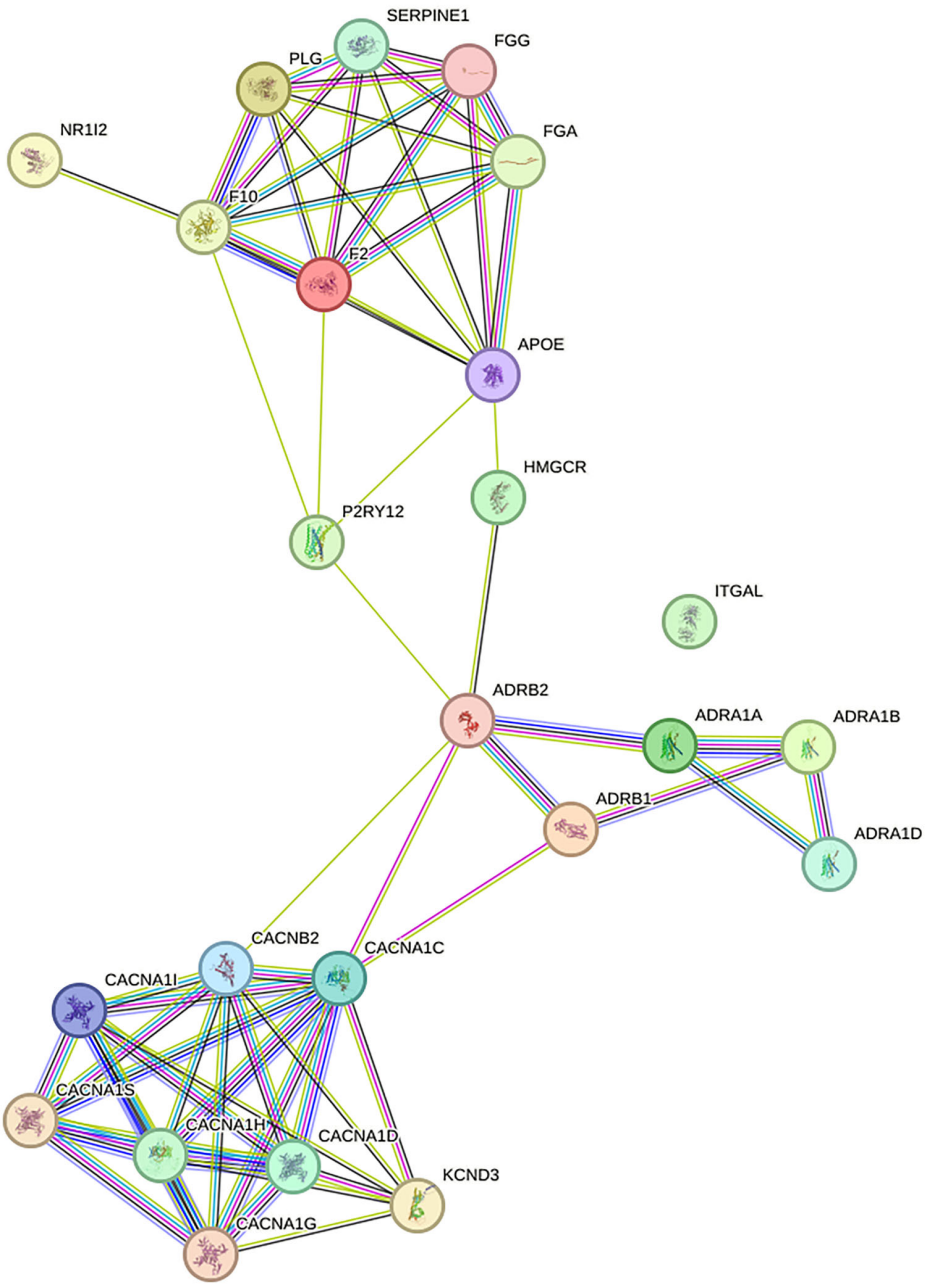
Protein–protein interaction (PPI) network analysis of existing drug targets and newly predicted targets for cerebral microbleeds. The network highlights key proteins targeted by currently used medications, including SERPINE1, F2, F10, PLG, FGG, P2RY12, FGA, APOE, and HMGCR.

To further identify potential therapeutic targets for CMBs, we examined eight drugs that are currently used for treatment (Supporting Information S5). Among the targets of these drugs, we identified several key proteins, including SERPINE1, F2, F10, PLG, FGG, P2RY12, FGA, and HMGCR (Figure 5). Given the hemorrhagic nature of CMBs, identifying these targets suggests a causal link between the coagulation pathway and CMB risk. These findings mechanistically align with the known side effects of anticoagulants and thrombolytics, rather than suggesting their repurposing for CMB treatment (Supporting Information S6).

### 3.4 The Impact of Lifestyle on Three Types of Proteins

**FIGURE 6:**
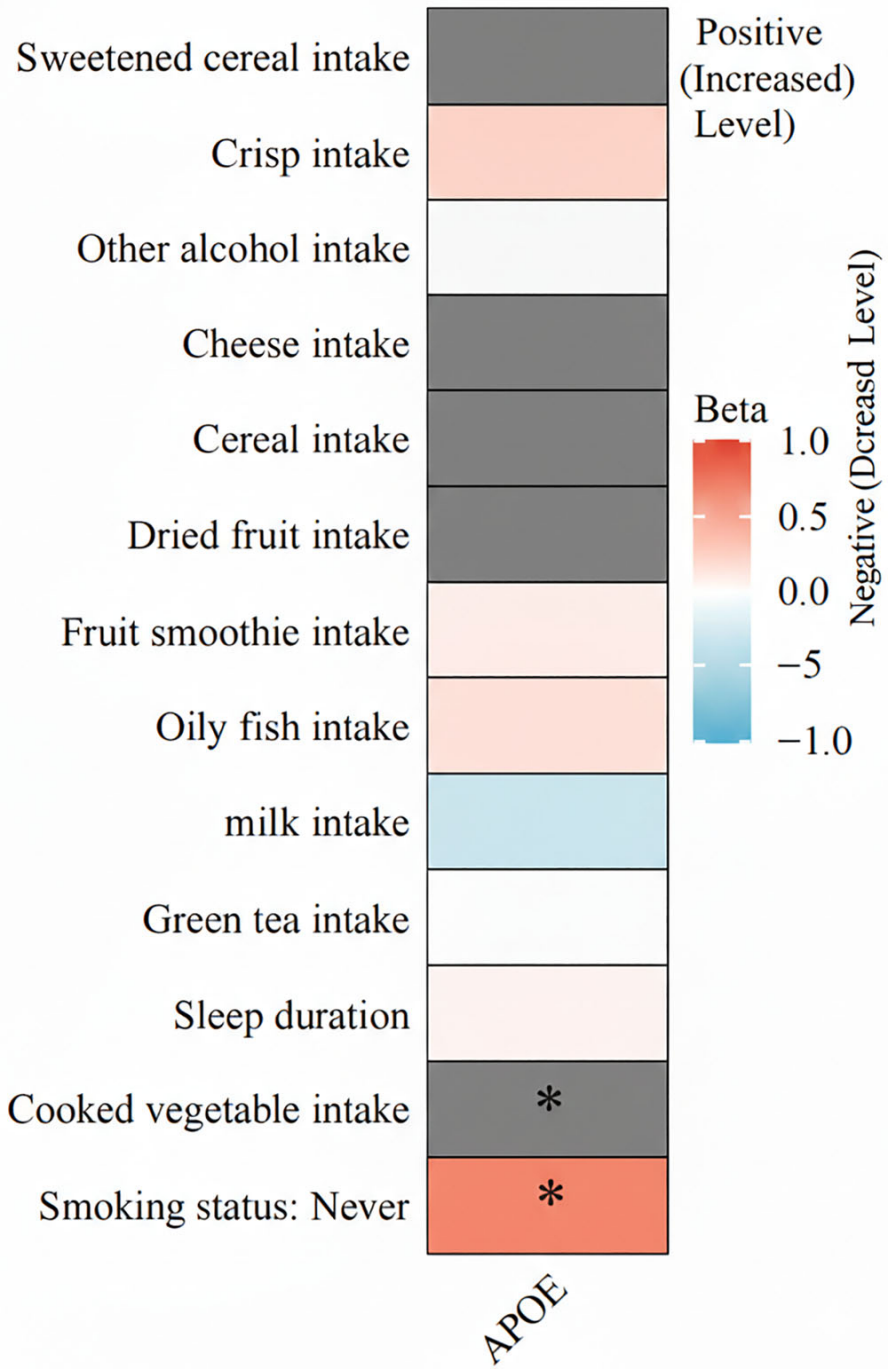
Heatmap illustrating the causal associations of lifestyle factors on APOE levels. This heatmap displays the Mendelian Randomization estimates for the association between 13 lifestyle factors and plasma APOE levels. The color scale on the right represents the direction and magnitude of the effect: Red indicates a positive association (an increase in the lifestyle factor is associated with higher APOE levels), while Blue indicates a negative association (lower APOE levels). The intensity of the color corresponds to the effect size. Asterisks (*) denote statistically significant associations (*P* < 0.05). Among the analyzed factors, Cooked vegetable intake showed a significant positive association with APOE levels, as indicated by the red shading and the asterisk. Other factors did not reach statistical significance in this analysis.

Of the 17 healthy lifestyle factors and one protein associated with CMBs that were analyzed using MR, only 13 lifestyle factors were included in the final analysis due to the use of APOE data located on chromosome 19. Among these factors, we found a significant association between cooked vegetable intake and APOE, suggesting that dietary habits, particularly vegetable consumption, may influence APOE‐related pathways involved in CMBs (Supporting Information S7, Figure 6).

## 4 Discussion

The present study provides novel insights into the complex relationship between circulating proteins and CMBs, leveraging genomic and proteomic approaches to elucidate potential causal mechanisms and therapeutic targets. Our comprehensive analysis, which integrated MR, colocalization analysis, and PPI network construction, has yielded several important findings with significant implications for understanding CMB pathogenesis and developing targeted interventions.

Our initial MR analysis of cis‐pQTLs and CMBs using UKB‐PPP data revealed associations between 79 plasma proteins and CMBs at a nominal significance level. However, the application of FDR correction, a crucial step in large‐scale proteomic studies, narrowed this list to a single protein that maintained statistical significance (Glickman et al. 2014). This stringent approach highlights the importance of rigorous statistical methods in proteome‐wide analyses to minimize false positives and ensure the robustness of identified associations. The subsequent validation of these findings using deCODE's cis‐pQTL data further strengthens the reliability of our results, demonstrating consistency across different datasets and populations (Gudbjartsson et al. 2015; Emilsson et al. 2008). While the association between the APOE ϵ4 allele and CMBs (via Cerebral Amyloid Angiopathy) is well‐established, our study validates this link using proteomic MR. This provides a distinct layer of evidence confirming that circulating APOE levels—regulated by cis‐variants—are causally linked to CMB risk, moving beyond simple genotype associations. The failure of the other 78 nominally significant proteins to pass FDR correction may reflect the high stringency of the analysis or limited power for proteins with smaller effect sizes. Nevertheless, these nominal associations (e.g., coagulation factors) provide valuable mechanistic insights.

The most striking finding of our study is the strong colocalization evidence for the APOE protein across multiple genomic windows, with a posterior probability (PP4) of 0.996. This result provides compelling evidence for a causal relationship between APOE and CMBs, a finding that aligns with and extends previous research on the role of APOE in cerebrovascular health (Coon et al. 2007). The Steiger test results further reinforced this causal link by confirming the directionality of the association, effectively ruling out reverse causality (Hemani et al. 2017). This robust evidence for APOE's causal role in CMB development has significant implications for both our understanding of CMB pathogenesis and potential therapeutic strategies.

The identification of APOE as a key player in CMB formation is particularly noteworthy given its well‐established role in lipid metabolism and its association with various neurodegenerative disorders, including Alzheimer's disease (C. C. Liu et al. 2013). Our findings suggest that the influence of APOE on CMB risk may be mediated through its effects on vascular integrity, potentially involving mechanisms such as blood–brain barrier dysfunction or alterations in cerebral amyloid deposition (Zlokovic 2011; Greenberg et al. 2020). This connection between APOE and CMBs provides a potential molecular link between vascular pathology and neurodegenerative processes, offering new avenues for investigating the complex interplay between these often co‐occurring phenomena. Evidence from systemic‐organ interaction studies suggests that circulating molecular traits can exert disease‐relevant effects on distal organs (K. Wang, Wang, Qin, et al. 2024; K. Wang, Wang, Chen, et al. 2024; Gao et al. 2023; K. Wang, Qin, et al. 2023).

From a clinical perspective, the strong association between APOE and CMBs underscores the potential utility of APOE genotyping in risk stratification for CMBs and related cerebrovascular events. Individuals carrying specific APOE alleles, particularly the ε4 allele known for its association with increased Alzheimer's risk, may benefit from more intensive monitoring and preventive strategies aimed at maintaining cerebrovascular health (Suri et al. 2015). Moreover, this finding suggests that therapeutic approaches targeting APOE or its downstream pathways could have far‐reaching implications for preventing or mitigating CMBs and their associated complications (Peng et al. 2019).

Our PPI network analysis identified proteins such as F2 (Thrombin), F10 (Factor X), PLG (Plasminogen), and SERPINE1. These are primary targets for anticoagulants (e.g., Rivaroxaban, Apixaban) and thrombolytics (e.g., Alteplase). Initially, one might consider these as potential therapeutic targets; however, the clinical reality of CMBs—which are prone to hemorrhage—dictates a different interpretation. Our results likely reflect the causal mechanism by which dysregulation (or inhibition) of these coagulation factors leads to CMBs. Consequently, the use of drugs inhibiting these factors is known to increase the risk of CMBs and intracerebral hemorrhage in clinical practice (Lioutas et al. 2019; Yokoyama et al. 2019). Therefore, rather than suggesting these drugs as treatments, our findings underscore the need for caution when prescribing anticoagulants to patients with a high burden of CMBs. This serves as a genetic validation of the bleeding risks associated with antithrombotic therapies.

The integration of lifestyle factors into our analysis provides a crucial link between genetic predisposition and modifiable risk factors for CMBs. Our MR analysis of 17 healthy lifestyle factors revealed a significant association between cooked vegetable intake and APOE levels. This finding is particularly intriguing as it suggests a potential pathway through which dietary habits may influence CMB risk via APOE‐related mechanisms. The positive association between vegetable consumption and APOE levels aligns with previous research on the beneficial effects of plant‐based diets on cardiovascular and cerebrovascular health (Gibbs et al. 2021; Li et al. 2018). However, the specific molecular pathways underlying this association require further investigation. This aligns with other MR studies on lifestyle exposures that urge nuanced interpretation of dietary impacts (Chen et al. 2024; K. Wang, Wang, Chen, and Lin 2024; S. Wang, Wang, Chen, Chen, et al. 2024).

This diet–gene interaction highlights the complex interplay between environmental factors and genetic predisposition in CMB development (Livingstone et al. 2018). It underscores the importance of considering both genetic and lifestyle factors in developing comprehensive strategies for CMB prevention and management. From a clinical perspective, this finding suggests that dietary interventions, particularly those emphasizing increased vegetable intake, could be an effective and accessible means of modulating CMB risk, especially in individuals with genetic susceptibility.

The broader implications of our study extend beyond CMBs to the wider field of cerebrovascular health. The identification of APOE as a causal factor for CMBs, combined with its known associations with other neurological disorders, suggests a common pathological pathway that may link various cerebrovascular and neurodegenerative conditions (Iadecola 2013; Kalaria 2016). This shared mechanism could explain the frequent co‐occurrence of CMBs with conditions such as Alzheimer's disease and vascular dementia, providing a new framework for understanding the spectrum of cerebrovascular pathologies (Ungvari et al. 2017).

Our findings also have significant implications for drug development and clinical trials. The proteins identified through our PPI network analysis, particularly those targeted by existing medications, represent promising candidates for further investigation. Future studies could explore the potential protective effects of drugs like statins (e.g., Rosuvastatin) or anticoagulants (e.g., Apixaban) on CMB formation and progression (Collins et al. 2016). However, it is crucial to approach such investigations with caution, considering the delicate balance between potential benefits and risks, especially given the hemorrhagic nature of CMBs.

The strong evidence for APOE's causal role in CMBs also opens up new avenues for personalized medicine approaches. Genetic testing for APOE variants could be incorporated into risk assessment protocols for cerebrovascular diseases, allowing for more targeted prevention strategies and earlier interventions (Shi et al. 2017). Moreover, this genetic information could be used to stratify patients in clinical trials, potentially leading to more efficient and effective evaluation of new therapies for CMBs and related conditions (Kamat et al. 2019).

While our study offers significant insights, it is essential to acknowledge its limitations. First, although the use of cis‐pQTLs as instrumental variables in our MR analysis mitigates the risk of pleiotropy, it may also constrain our capacity to detect associations with proteins that are regulated by trans‐acting genetic variants (Westra et al. 2013). Second, focusing solely on plasma proteins may not adequately represent the intricate protein interactions within the brain microenvironment where CMBs occur (Henneberger et al. 2020). Future studies that incorporate cerebrospinal fluid proteomics or advanced neuroimaging techniques are warranted to better elucidate the local factors contributing to CMB formation (Siddiqi et al. 2019). Additionally, while our findings indicate a significant association between vegetable intake and APOE levels, a more comprehensive understanding of how various lifestyle factors interact to influence CMB risk remains to be fully elucidated. Longitudinal studies investigating the impact of comprehensive lifestyle interventions on CMB incidence and progression will be vital for translating these findings into effective preventive strategies.

In conclusion, our research represents a significant advancement in understanding the molecular mechanisms underlying CMBs. By integrating advanced genomic and proteomic methodologies with an analysis of lifestyle factors, we have identified APOE as a critical causal factor in CMB development and highlighted potential therapeutic targets as well as lifestyle interventions. These findings not only deepen our understanding of CMB pathogenesis but also pave the way for more targeted and personalized approaches to prevention and treatment. As we continue to unravel the complex interplay between genetic, proteomic, and environmental factors in cerebrovascular health, we move closer to developing effective strategies to alleviate the burden of CMBs and their associated neurological complications. Future research building on these findings has the potential to significantly influence clinical practice and improve outcomes for individuals at risk of cerebrovascular disease.

## Author Contributions

**Lei Yang**: conceptualization, methodology, writing – original draft. **Jian‐Lan Zhao**: data curation, investigation. **Jie Song**: formal analysis, methodology, validation. **Lin‐Hui Chen**: formal analysis, methodology, validation. **Chun Yu**: formal analysis, methodology, validation. **Qiang Yuan**: writing – review and editing, validation. **Gang Wu**: resources, software, supervision, project administration. **Jin Hu**: resources, software, supervision, project administration, funding acquisition. **Mei‐Hua Wang**: resources, software, supervision, project administration.

## Funding

This study was supported by the National Natural Science Foundation of China (Nos. 82471407 and 82171382) and the Shanghai Shenkang Hospital Development Center, Medical Technology Promotion and Optimization Management project for municipal hospitals (SHDC22022210).

## Ethics Statement

The authors have nothing to report.

## Consent

The authors have nothing to report.

## Conflicts of Interest

The authors declare no conflicts of interest.

## Supporting information




**Supplementary Material**: brb371382‐sup‐0001‐SuppMat.xlsx

**Supplementary Material**: brb371382‐sup‐0002‐SuppMat.xlsx

**Supplementary Material**: brb371382‐sup‐0003‐SuppMat.xlsx

**Supplementary Material**: brb371382‐sup‐0004‐SuppMat.xlsx

**Supplementary Material**: brb371382‐sup‐0005‐SuppMat.xlsx

**Supplementary Material**: brb371382‐sup‐0006‐SuppMat.xlsx

**Supplementary Material**: brb371382‐sup‐0007‐SuppMat.xlsx

## Data Availability

The datasets used and/or analyzed during the current study are available from the corresponding author on reasonable request.

## References

[brb371382-bib-0001] Greenberg, S. M. , M. W. Vernooij , C. Cordonnier , et al. 2009. “Cerebral Microbleeds: A Guide to Detection and Interpretation.” Lancet Neurology 8, no. 2: 165–174. 10.1016/S1474-4422(09)70013-4. 19161908 PMC3414436

[brb371382-bib-0002] Charidimou, A. , G. Boulouis , M. E. Gurol , et al. 2017. “Emerging Concepts in Sporadic Cerebral Amyloid Angiopathy.” Brain 140, no. 7: 1829–1850. 10.1093/brain/awx047. 28334869 PMC6059159

[brb371382-bib-0003] Wardlaw, J. M. , E. E. Smith , G. J. Biessels , et al. 2013. “Neuroimaging Standards for Research Into Small Vessel Disease and Its Contribution to Ageing and Neurodegeneration.” Lancet Neurology 12, no. 8: 822–838. 10.1016/S1474-4422(13)70124-8. 23867200 PMC3714437

[brb371382-bib-0004] Shams, S. , J. Martola , T. Granberg , et al. 2015. “Cerebral Microbleeds: Different Prevalence, Topography, and Risk Factors Depending on Dementia Diagnosis—A Population‐based Study.” American Journal of Neuroradiology 36, no. 4: 661–666. 10.3174/ajnr.A4176. 25523590 PMC7964321

[brb371382-bib-0005] Poels, M. M. F. , M. A. Ikram , A. Van Der Lugt , et al. 2011. “Incidence of Cerebral Microbleeds in the General Population: the Rotterdam Scan Study.” Stroke 42, no. 3: 656–661. 10.1161/STROKEAHA.110.607184. 21307170

[brb371382-bib-0006] Jolink, W. M. T. , S. J. Van Veluw , J. J. M. Zwanenburg , et al. 2023. “Histopathology of Cerebral Microinfarcts and Microbleeds in Spontaneous Intracerebral Hemorrhage.” Translational Stroke Research 14, no. 2: 174–184. 10.1007/s12975-022-01016-5. 35384634 PMC9995541

[brb371382-bib-0007] Shim, Y. S. , D.‐W. Yang , C. M. Roe , et al. 2015. “Pathological Correlates of White Matter Hyperintensities on Magnetic Resonance Imaging.” Dementia and Geriatric Cognitive Disorders 39, no. 1–2: 92–104. 10.1159/000366411. 25401390 PMC4312498

[brb371382-bib-0008] Akoudad, S. , M. L. P. Portegies , P. J. Koudstaal , et al. 2015. “Cerebral Microbleeds Are Associated With an Increased Risk of Stroke: the Rotterdam Study.” Circulation 132, no. 6: 509–516. 10.1161/CIRCULATIONAHA.115.016261. 26137955

[brb371382-bib-0009] Tan, R. Y. , and H. S. Markus . 2015. “Genetic Associations With Sporadic Cerebral Small Vessel Disease.” Stroke 46, no. 9: 2712–2724.

[brb371382-bib-0010] Söderholm, M. , A. Pedersen , E. Lorentzen , et al. 2019. “Genome‐Wide Association Meta‐Analysis of Functional Outcome After Ischemic Stroke.” Neurology 92, no. 12: e1271–e1283. 30796134 10.1212/WNL.0000000000007138 PMC6511098

[brb371382-bib-0011] Dutta, P. , G. Courties , Y. Wei , et al. 2012. “Myocardial Infarction Accelerates Atherosclerosis.” Nature 487, no. 7407: 325–329. 10.1038/nature11260. 22763456 PMC3401326

[brb371382-bib-0012] Davey Smith, G. , and G. Hemani . 2014. “Mendelian Randomization: Genetic Anchors for Causal Inference in Epidemiological Studies.” Human Molecular Genetics 23, no. R1: R89–R98. 10.1093/hmg/ddu328. 25064373 PMC4170722

[brb371382-bib-0013] Emdin, C. A. , A. V. Khera , and S. Kathiresan . 2017. “Mendelian Randomization.” Jama 318, no. 19: 1925. 10.1001/jama.2017.17219. 29164242

[brb371382-bib-0014] Raffield, L. M. , A. T. Lu , M. D. Szeto , et al. 2020. “Coagulation Factor VIII: Relationship to Cardiovascular Disease Risk and Whole Genome Sequence and Epigenome‐Wide Analysis in African Americans.” Journal of Thrombosis and Haemostasis 18, no. 6: 1335–1347. 10.1111/jth.14741. 31985870 PMC7274883

[brb371382-bib-0015] Zhang, M. , S. Serna‐Salas , T. Damba , M. Borghesan , M. Demaria , and H. Moshage . 2021. “Hepatic Stellate Cell Senescence in Liver Fibrosis: Characteristics, Mechanisms and Perspectives.” Mechanisms of Ageing and Development 199: 111572. 10.1016/j.mad.2021.111572. 34536446

[brb371382-bib-0016] Liu, Y. , C. Shen , and Y. Cao . 2025. “Mediating Role of Blood Metabolites in the Relationship between Immune Cell Traits and Heart Failure: A Mendelian Randomization and Mediation Analysis.” Journal of the American Heart Association 14, no. 6:e037265. 10.1161/JAHA.124.037265. 40079309 PMC12132714

[brb371382-bib-0017] Nian, S. , K. Wang , J. Wang , et al. 2025. “Causal Associations Between Immune Cell Phenotypes and Varicose Veins: A Mendelian Randomization Analysis.” Annals of Vascular Surgery 114: 126–132. 10.1016/j.avsg.2025.01.030. 39884498

[brb371382-bib-0018] Liu, R. , K. Wang , X. Guo , et al. 2024. “A Causal Relationship Between Distinct Immune Features and Acute or Chronic Pancreatitis: Results From a Mendelian Randomization Analysis.” Pancreatology 24, no. 8: 1219–1228. 10.1016/j.pan.2024.10.006. 39419750

[brb371382-bib-0019] Hu, Y. , K. Wang , Y. Chen , Y. Jin , Q. Guo , and H. Tang . 2024. “Causal Relationship Between Immune Cell Phenotypes and Risk of Biliary Tract Cancer: Evidence From Mendelian Randomization Analysis.” Frontiers in Immunology 15: 1430551. 10.3389/fimmu.2024.1430551. 39050844 PMC11266158

[brb371382-bib-0020] Evangelou, E. , and J. P. A. Ioannidis . 2013. “Meta‐Analysis Methods for Genome‐Wide Association Studies and Beyond.” Nature Reviews Genetics 14, no. 6: 379–389. 10.1038/nrg3472. 23657481

[brb371382-bib-0021] Wardlaw, J. , E. E. Smith , and M. Dichgans . 2017. “Small Vessel Disease: Mechanisms and Clinical Implications.” Lancet Neurology 16, no. 8: 790–801. 10.1016/S1474-4422(19)30079-1 31097385

[brb371382-bib-0022] Kim, Y. , J. H. Park , and Y. R. Cho . 2022. “Network‐Based Approaches for Disease‐Gene Association Prediction Using Protein‐Protein Interaction Networks.” International Journal of Molecular Sciences 23, no. 13: 7411. 10.3390/ijms23137411. 35806415 PMC9266751

[brb371382-bib-0023] Emilsson, V. , M. Ilkov , J. R. Lamb , et al. 2018. “Co‐Regulatory Networks of Human Serum Proteins Link Genetics to Disease.” Science 361, no. 6404: 769–773. 10.1126/science.aaq1327. 30072576 PMC6190714

[brb371382-bib-0024] Sun, B. B. , J. C. Maranville , J. E. Peters , et al. 2018. “Genomic Atlas of the Human Plasma Proteome.” Nature 558, no. 7708: 73–79. 10.1038/s41586-018-0175-2. 29875488 PMC6697541

[brb371382-bib-0025] Zheng, J. , V. Haberland , D. Baird , et al. 2020. “Phenome‐Wide Mendelian Randomization Mapping the Influence of the Plasma Proteome on Complex Diseases.” Nature Genetics 52, no. 10: 1122–1131. 10.1038/s41588-020-0682-6. 32895551 PMC7610464

[brb371382-bib-0026] Yaghootkar, H. , R. C. Richmond , W. Spiller , et al. 2020. “Genetic Evidence for a Normal‐Weight “Metabolically Obese” Phenotype Linking Insulin Resistance, Hypertension, Coronary Artery Disease, and Type 2 Diabetes.” Diabetes 69, no. 11: 2447–2457. 10.2337/db14-0318 PMC4392920 25048195

[brb371382-bib-0027] Burgess, S. , and S. G. Thompson . 2015. Mendelian Randomization: Methods for Using Genetic Variants in Causal Estimation. Chapman and Hall/CRC. 10.1201/b18084.

[brb371382-bib-0028] Bowden, J. , G. Davey Smith , P. C. Haycock , and S. Burgess . 2016. “Consistent Estimation in Mendelian Randomization With Some Invalid Instruments Using a Weighted Median Estimator.” Genetic Epidemiology 40, no. 4: 304–314. 10.1002/gepi.21965. 27061298 PMC4849733

[brb371382-bib-0029] Giambartolomei, C. , D. Vukcevic , E. E. Schadt , et al. 2014. “Bayesian Test for Colocalisation Between Pairs of Genetic Association Studies Using Summary Statistics.” PLOS Genetics 10, no. 5: e1004383. 10.1371/journal.pgen.1004383. 24830394 PMC4022491

[brb371382-bib-0030] Wallace, C. 2020. “Eliciting Priors and Relaxing the Single Causal Variant Assumption in Colocalisation Analyses.” PLOS Genetics 16, no. 4: e1008720. 10.1371/journal.pgen.1008720. 32310995 PMC7192519

[brb371382-bib-0031] Wishart, D. S. , and A. Wu . 2016. “Using DrugBank for in Silico Drug Exploration and Discovery.” Current Protocols in Bioinformatics 54, no. 1: 14.4.1–14.4.31. 10.1002/cpbi.1. 27322405

[brb371382-bib-0032] Liu, Y. , A. Buil , B. C. Collins , et al. 2019. “Quantitative Variability of 24,000 Human Proteins in Plasma From the UK Biobank.” Nature 562, no. 7727: 82–88.

[brb371382-bib-0033] Glickman, M. E. , S. R. Rao , and M. R. Schultz . 2014. “False Discovery Rate Control Is a Recommended Alternative to Bonferroni‐Type Adjustments in Health Studies.” Journal of Clinical Epidemiology 67, no. 8: 850–857. 10.1016/j.jclinepi.2014.03.012. 24831050

[brb371382-bib-0034] Gudbjartsson, D. F. , H. Helgason , S. A. Gudjonsson , et al. 2015. “Large‐Scale Whole‐Genome Sequencing of the Icelandic Population.” Nature Genetics 47, no. 5: 435–444. 10.1038/ng.3247. 25807286

[brb371382-bib-0035] Emilsson, V. , G. Thorleifsson , B. Zhang , et al. 2008. “Genetics of Gene Expression and Its Effect on Disease.” Nature 452, no. 7186: 423–428. 10.1038/nature06758. 18344981

[brb371382-bib-0036] Coon, K. D. , A. J. Myers , D. W. Craig , et al. 2007. “A High‐Density Whole‐Genome Association Study Reveals That APOE Is the Major Susceptibility Gene for Sporadic Late‐Onset Alzheimer's Disease.” Journal of Clinical Psychiatry 68, no. 4: 613–618. 10.4088/JCP.v68n0419. 17474819

[brb371382-bib-0037] Hemani, G. , K. Tilling , and G. Davey Smith . 2017. “Orienting the Causal Relationship Between Imprecisely Measured Traits Using GWAS Summary Data.” PLOS Genetics 13, no. 11: e1007081. 10.1371/journal.pgen.1007081. 29149188 PMC5711033

[brb371382-bib-0038] Liu, C. C. , T. Kanekiyo , H. Xu , and G. Bu . 2013. “Apolipoprotein E and Alzheimer Disease: Risk, Mechanisms, and Therapy.” Nature Reviews Neurology 9, no. 2: 106–118. 10.1038/nrneurol.2012.263. 23296339 PMC3726719

[brb371382-bib-0039] Zlokovic, B. V. 2011. “Neurovascular Pathways to Neurodegeneration in Alzheimer's Disease and Other Disorders.” Nature Reviews Neuroscience 12, no. 12: 723–738. 10.1038/nrn3114. 22048062 PMC4036520

[brb371382-bib-0040] Greenberg, S. M. , B. J. Bacskai , M. Hernandez‐Guillamon , J. Pruzin , R. Sperling , and S. J. Van Veluw . 2020. “Cerebral Amyloid Angiopathy and Alzheimer Disease—One Peptide, Two Pathways.” Nature Reviews Neurology 16, no. 1: 30–42. 10.1038/s41582-019-0281-2. 31827267 PMC7268202

[brb371382-bib-0041] Wang, K. , S. Wang , X. Qin , et al. 2024. “The Causal Relationship Between Gut Microbiota and Biliary Tract Cancer: Comprehensive Bidirectional Mendelian Randomization Analysis.” Frontiers in Cellular and Infection Microbiology 14: 1308742. 10.3389/fcimb.2024.1308742. 38558852 PMC10978781

[brb371382-bib-0042] Wang, K. , S. Wang , Y. Chen , et al. 2024. “Causal Relationship Between Gut Microbiota and Risk of Gastroesophageal Reflux Disease: A Genetic Correlation and Bidirectional Mendelian Randomization Study.” Frontiers in Immunology 15: 1327503. 10.3389/fimmu.2024.1327503. 38449873 PMC10914956

[brb371382-bib-0043] Gao, X. , Z. Wang , B. Liu , and Y. Cheng . 2023. “Causal Association of Gut Microbiota and Esophageal Cancer: A Mendelian Randomization Study.” Frontiers in Microbiology 14: 1286598. 10.3389/fmicb.2023.1286598. 38107856 PMC10722290

[brb371382-bib-0044] Wang, K. , X. Qin , T. Ran , et al. 2023. “Causal Link Between Gut Microbiota and Four Types of Pancreatitis: A Genetic Association and Bidirectional Mendelian Randomization Study.” Frontiers in Microbiology 14: 1290202. 10.3389/fmicb.2023.1290202. 38075894 PMC10702359

[brb371382-bib-0045] Suri, S. , A. Topiwala , N. Filippini , et al. 2015. “APOE ε4 Genotype and Cerebral Blood Flow in Young Adults: A Cross‐Sectional Study.” Lancet Neurology 14, no. 8: 873–881.

[brb371382-bib-0046] Peng, Y. , J. Xu , Y. Zeng , L. Chen , and X. L. Xu . 2019. “Polydatin Attenuates Atherosclerosis in Apolipoprotein E‐Deficient Mice: Role of Reverse Cholesterol Transport.” Phytomedicine 62: 152935. 10.1016/j.phymed.2019.152935. 31085374

[brb371382-bib-0047] Lioutas, V. A. , N. Goyal , A. H. Katsanos , et al. 2019. “Microbleed Prevalence and Burden in Anticoagulant‐Associated Intracerebral Bleed.” Annals of Clinical and Translational Neurology 6, no. 8: 1546–1551. 10.1002/acn3.50834. 31402613 PMC6689674

[brb371382-bib-0048] Yokoyama, M. , A. Mizuma , T. Terao , et al. 2019. “Effectiveness of Nonvitamin K Antagonist Oral Anticoagulants and Warfarin for Preventing Further Cerebral Microbleeds in Acute Ischemic Stroke Patients With Nonvalvular Atrial Fibrillation and at Least One Microbleed: CMB‐NOW Multisite Pilot Trial.” Journal of Stroke and Cerebrovascular Diseases 28, no. 7: 1918–1925. 10.1016/j.jstrokecerebrovasdis.2019.03.050. 31005561

[brb371382-bib-0049] Gibbs, J. , E. Gaskin , C. Ji , M. A. Miller , and F. P. Cappuccio . 2021. “The Effect of Plant‐based Dietary Patterns on Blood Pressure: A Systematic Review and Meta‐Analysis of Controlled Intervention Trials.” Journal of Hypertension 39, no. 1: 23–37. 10.1097/HJH.0000000000002604. 33275398

[brb371382-bib-0050] Li, Y. , A. Pan , D. D. Wang , et al. 2018. “Impact of Healthy Lifestyle Factors on Life Expectancies in the US Population.” Circulation 138, no. 4: 345–355. 10.1161/CIRCULATIONAHA.117.032047. 29712712 PMC6207481

[brb371382-bib-0051] Chen, Y. , L. Yang , K. Wang , et al. 2024. “Relationship Between Fatty Acid Intake and Aging: A Mendelian Randomization Study.” Aging 16, no. 6: 5711–5739. 10.18632/aging.205674. 38535988 PMC11006485

[brb371382-bib-0052] Wang, S. , K. Wang , X. Chen , and S. Lin . 2024. “The Relationship Between Autoimmune Thyroid Disease, Thyroid Nodules and Sleep Traits: A Mendelian Randomization Study.” Frontiers in Endocrinology 14: 1325538. 10.3389/fendo.2023.1325538. 38562570 PMC10982365

[brb371382-bib-0053] Wang, S. , K. Wang , X. Chen , D. Chen , and S. Lin . 2024. “Autoimmune Thyroid Disease and Myasthenia Gravis: A Study Bidirectional Mendelian Randomization.” Frontiers in Endocrinology 15: 1310083. 10.3389/fendo.2024.1310083. 38405140 PMC10884276

[brb371382-bib-0054] Livingstone, K. M. , C. Celis‐Morales , J. Lara , et al. 2018. “Associations Between Adherence to Healthy Dietary Guidelines and Anthropometric Measures and Cardiovascular Risk Factors in the UK Biobank Study.” American Journal of Clinical Nutrition 107, no. 6: 919–930.

[brb371382-bib-0055] Iadecola, C. 2013. “The Pathobiology of Vascular Dementia.” Neuron 80, no. 4: 844–866. 10.1016/j.neuron.2013.10.008. 24267647 PMC3842016

[brb371382-bib-0056] Kalaria, R. N. 2016. “Neuropathological Diagnosis of Vascular Cognitive Impairment and Vascular Dementia With Implications for Alzheimer's Disease.” Acta Neuropathologica 131, no. 5: 659–685. 10.1007/s00401-016-1571-z. 27062261 PMC4835512

[brb371382-bib-0057] Ungvari, Z. , S. Tarantini , A. C. Kirkpatrick , A. Csiszar , and C. I. Prodan . 2017. “Cerebral Microhemorrhages: Mechanisms, Consequences, and Prevention.” American Journal of Physiology‐Heart and Circulatory Physiology 312, no. 6: H1128–H1143. 10.1152/ajpheart.00780.2016. 28314762 PMC5495931

[brb371382-bib-0058] Collins, R. , C. Reith , J. Emberson , et al. 2016. “Interpretation of the Evidence for the Efficacy and Safety of Statin Therapy.” Lancet 388, no. 10059: 2532–2561. 10.1016/S0140-6736(16)31357-5. 27616593

[brb371382-bib-0059] Shi, Y. , K. Yamada , S. A. Liddelow , et al. 2017. “ApoE4 Markedly Exacerbates Tau‐Mediated Neurodegeneration in a Mouse Model of Tauopathy.” Nature 549, no. 7673: 523–527. 10.1038/nature24016. 28959956 PMC5641217

[brb371382-bib-0060] Kamat, M. A. , J. A. Blackshaw , R. Young , et al. 2019. “PhenoScanner V2: An Expanded Tool for Searching Human Genotype‐Phenotype Associations.” Bioinformatics 35, no. 22: 4851–4853. 10.1093/bioinformatics/btz469. 31233103 PMC6853652

[brb371382-bib-0061] Westra, H.‐J. , M. J. Peters , T. Esko , et al. 2013. “Systematic Identification of Trans eQTLs as Putative Drivers of Known Disease Associations.” Nature Genetics 45, no. 10: 1238–1243. 10.1038/ng.2756. 24013639 PMC3991562

[brb371382-bib-0062] Henneberger, C. , L. Bard , A. Panatier , et al. 2020. “LTP Induction Boosts Glutamate Spillover by Driving Withdrawal of Perisynaptic Astroglia.” Neuron 108, no. 5: 919–936.e11. 10.1016/j.neuron.2020.08.030. 32976770 PMC7736499

[brb371382-bib-0063] Siddiqi, M. K. , S. Malik , N. Majid , P. Alam , and R. H. Khan . 2019. “Cytotoxic Species in Amyloid‐Associated Diseases: Oligomers or Mature Fibrils.” Advances in Protein Chemistry and Structural Biology 118: 333–369. 31928731 10.1016/bs.apcsb.2019.06.001

